# Achieving efficient clonal beta cells transfection using nanostraw/nanopore-assisted electroporation†

**DOI:** 10.1039/d4ra02791d

**Published:** 2024-07-15

**Authors:** Frida Ekstrand, Mokhtar Mapar, Sabrina Ruhrmann, Karl Bacos, Charlotte Ling, Christelle N. Prinz

**Affiliations:** a Division of Solid State Physics, NanoLund, Lund University 221 00 Lund Sweden christelle.prinz@ftf.lth.se; b Epigenetics and Diabetes Unit, Lund University Diabetes Centre, Department of Clinical Sciences, Scania University Hospital 214 28 Malmö Sweden

## Abstract

The prospect of being able to efficiently inject large plasmids in insulin-producing beta cells is very attractive for diabetes research. However, conventional transfection methods suffer from high cytotoxicity or low transfection efficiency, which negatively affect their outcome. In contrast, nanostraw electroporation is a gentle method that can provide a high transfection efficiency while maintaining high cell viability. While nanostraw electroporation has gone through some method optimization in the past, such as tuning the pulse frequency, amplitude, and duration, the effect of other parameters has not been thoroughly investigated. Here, we demonstrate efficient transfection of clonal beta cells and investigate the effect of voltage at a fixed inter-electrode distance, cell density, and cargo solution conductivity on transfection efficiency. We used GFP-encoding DNA plasmids stained with an intercalating dye to enable immediate analysis and assessment of the electrophoretic transport of cargo. Moreover, we ran simulations to assess how cargo buffer conductivity impacts the transfection efficiency by affecting the voltage drop on the nanostraws and cell membrane during electroporation. Both experiments and simulations show that MilliQ water as the cargo buffer yields the best transfection efficiency. We also show that the cell density should be adjusted to maximize the number of cells interfacing the nanostraws and avoid cell stacking. Finally, we compared the transfection efficiency when using nanostraws and nanopores. Whereas the amount of GFP plasmids injected using nanostraws is larger than for nanopores, the outcome in terms of GFP fluorescence 48 h after transfection was worse than for nanopores. Moreover, when using nanostraws, fewer cells were found on the substrate 48 h after transfection compared to when using nanopores. This suggests that injecting substantial amounts of plasmids in cells can affect their proliferation and/or viability, and that nanopore electroporation, as a simpler method, is an interesting alternative to nanostraws in achieving efficient and gentle clonal beta cell transfection.

## Abstract 2

GFP plasmids are injected in cells using nanostraw and nanopore-electroporation. After optimization, the results show that more plasmids are delivered in cells using nanostraws, but fewer cells express GFP 48 h after injection, compared to nanopores.

## Graphical abstract

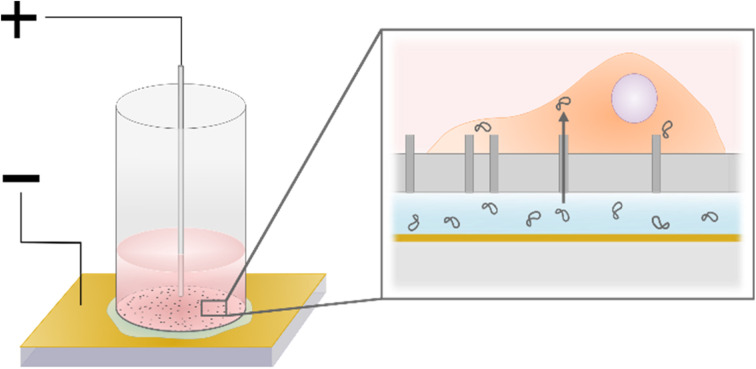

## Introduction

Enabling the transfection of beta cells with high efficiency would open the possibility for genetic and epigenetic corrections and possibly new therapeutic avenues in diabetes research. For instance, one could specifically induce or correct previously identified unfavorable genetic or epigenetic traits to either mimic or correct disease characteristics, such as insulin production or secretion, in beta cells. Conventional transfection methods such as viruses and lipofection suffer from problems such as mutagenesis^1^ and endosomal entrapment,^2^ respectively. Nanostraw electroporation (NS-EP) is another method increasingly used to transfect cells. It has been used on a variety of cell types, and the advantage of this method lies in its superior transfection efficiency and high cell viability.^3–6^ For instance, comparing two independent studies using lipofection^7^ and NS-EP^8^ to transfect GFP plasmids in HeLa cells reveals that NS-EP resulted in three-fold higher transfection efficiency than when using lipofection, with similar cell viability. Moreover, several studies show that sublethal damage, such as effects on cell proliferation and gene expression, is minimal when using NS-EP.^6,9^

The NS-EP method consists of seeding or spinning down cells on a nanostraw substrate with cargo in solution (such as DNA,^10^ RNA,^11^ proteins,^12^ or nanoparticles^13^) on the other side of the substrate. The cargo injection is achieved *via* the application of electrical pulses across the nanostraw substrate, which (i) locally opens the cell membrane on top of the nanostraws,^14^ and (ii) drives the cargo through the nanostraws to the cytosol using electrophoresis.^4^

Whereas the general principles of the method NS-EP are well understood, the method optimization was undertaken only recently, with investigations of the effect of pulse amplitude, duration, and repetition frequency on viability and efficiency.^9,12^ Although these studies are valuable, the role of various parameters on transfection efficiency is still not fully understood. For instance, some studies vary the voltage applied between electrodes without mentioning the distance between electrodes, which prevents the evaluation of the electric field strengths and the comparison of results between studies. Regarding cargo choice, propidium iodide (PI) has often been used as model cargo as it cannot cross an intact cell membrane.^15^ Therefore, the presence of PI in the cytosol after NS-EP was interpreted as the signature of a successful nanostraw-induced cargo injection. However, PI is a small molecule with rapid diffusion kinetics. It can readily diffuse across the nanostraws within ≈1 s and enter cells even in the absence of electrophoretic forces. Moreover, small molecules have been reported to enter cells spontaneously because of membrane curvature and membrane stress.^16^ As a result, assessing the electrophoretic transport efficiency in studies using PI is not straightforward, and distinguishing diffusion from electrophoretic transport can be challenging. Using large molecules with slow diffusion kinetics (within the experimental time frame), such as large DNA plasmids coding for fluorescent proteins, ensures that electrophoresis is the primary cargo transport mechanism across the nanostraw substrate and, therefore, the main contributor to the transfection efficiency after cell membrane electroporation.^17^ However, using these plasmids requires waiting 24 hours before assessing the transfection success, which can then be affected by cell division.

Another issue is that most studies use fluorescence microscopy to evaluate NS-EP efficiency. This requires an area selection and makes it difficult to assess all cells on the substrate.^15,18^ Using flow cytometry instead, all cells on the nanostraw substrate, irrespective of their position on the substrate, can be included in the assessment of transfection efficiency. To obtain reliable results from flow cytometry, a high cell count is needed. This depends on the cell density on the nanostraw substrate, which is an important factor that can possibly affect the transfection efficiency. When this was investigated on HeLa cells,^12^ no clear effect was identified for densities up to 2200 cells per mm^2^. However, higher cell densities were not investigated.

Also not fully understood is the effect of the cargo-solution ionic strength on NS-EP. Previous studies have used different ionic strength buffers as cargo solutions (ranging from deionized water^4^ to physiological condition buffers^12^), however, the rationale behind these choices is unclear. The effect of various parameters, such as membrane tension, molecular diffusion, and electric field, have been investigated by simulations.^9,10,19,20^ However, so far, variations related to buffer conductivity have not been studied. Finally, the effect of straw length has been explored to some extent.^6^ There are also a few promising studies where nanopores (straw length 0 μm) have been used to transfect cells,^8,21^ which are easier to fabricate and a cheaper alternative to nanostraws.

Here, we demonstrate efficient transfection of clonal beta cells using NS-EP. We used simulations in parallel with experiments to shed light on how varying the electrode voltage, cell density, cargo-solution ionic strength, and nanostraw length affect the transfection efficiency for a fixed electrode distance. We used GFP-encoded DNA plasmids fluorescently labeled with an intercalating dye as a cargo, which enabled us to assess membrane electroporation, cargo electrophoresis, and the resulting transfection efficiency, immediately after transfection. We used flow cytometry as a read-out method, ensuring that every cell on the nanostraw substrate was included in the assay. Finally, we provide a comprehensive understanding of the effect of the cargo solution's ionic strength on membrane electroporation and cargo electrophoresis.

## Experimental

### Nanostraw/nanopore substrate fabrication

**Fig. 1:**
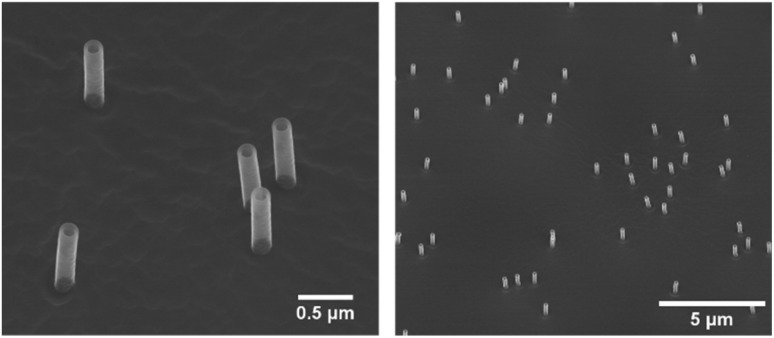
Scanning electron microscope (SEM) images of nanostraws, with a height of 1 μm and outer diameter of 160 nm. In-lens detector, 30° tilt.

Nanostraw substrates were made from track-etched polycarbonate (PC) membranes, with a thickness of 25 μm, pore density of 2 × 10^7^ cm^−2^, and nominal pore diameter of 200 nm (it4ip, Belgium). The PC membranes were coated with ≈12 nm of alumina, using atomic layer deposition (ALD, Savannah, Cambridge Nanotech) at 90 °C with alternating pulses of trimethylaluminum and H_2_O (130 cycles of 0.15 s long pulses with 30 s inter-pulse pumping time). The coated membranes were then fixed on a 4′′ silicon wafer using an anti-static gun (Zerostat, VWR) to allow sufficient heat transfer during etching. Inductively coupled plasma and reactive ion etching (ICP-RIE, APEX SLR Advanced Vacuum Systems AB) was subsequently performed in two steps; (1) ICP-RIE using argon at 40 sccm for 2.5 minutes, with RIE set to 60 W and ICP to 400 W, resulting in the removal of alumina from the horizontal surfaces of the PC membrane, (2) ICP-RIE using SF_6_ at five sccm and O_2_ at 45 sccm for ≈1.5 minutes, with RIE and ICP set to 50 W and 400 W, respectively, resulting in the removal of ≤1 μm of PC. Both steps had helium cooling with a flow of 5 sccm. The whole process resulted in alumina nanostraws protruding from the PC membrane (0.5–1 μm in height and 160 ± 10 nm in outer diameter). After fabrication, each nanostraw substrate was imaged using a scanning electron microscope (SEM, LEO Gemini 1560, LEO Electron Microscopy, Inc.), see Fig. 1. Before SEM imaging, a piece of the nanostraw substrate was fixed on an SEM stud using carbon tape and subsequently sputter coated with 5–10 nm of Pt : Pd (80 : 20) or Ir (Q150T ES sputter coater, Quorum Technologies).

Nanopore substrates were prepared in the exact same way as the nanostraw substrates, however without the etching steps.

### Cell culture

The cell type used for all experiments in this paper was clonal beta cells (832/13 INS-1 rat insulinoma cells)^22^ with a population doubling number 24-60. Beta cells are insulin-producing cells; the prospective of being able to inject large plasmids in these cells is very attractive for diabetes research, justifying this choice of cells for maximizing nanostraw transfection. For culturing, RPMI-1640 culture medium (SH30027.01, Cytiva, HyClone) was supplemented with 10% heat-inactivated fetal bovine serum (FBS, qualified, Brazil origin, Gibco), 1% penicillin-streptomycin (Sigma Aldrich), and 2.2% supplement (50% glutamine solution 200 mM (Gibco), 50% sodium pyruvate solution 100 mM (Gibco), 176 ppm 2-mercaptoethanol), hereafter called full medium. Cells were cultured at 37 °C under a 5% CO_2_ atmosphere and split when reaching ≈80–100% confluency. For passaging, cells were rinsed with 1× Dulbecco's phosphate-buffered saline (DPBS, SH30028.FS, Thermo Fisher) before incubation with trypsin-EDTA (Gibco) for 3 minutes. Trypsinization was interrupted by adding full medium, and the cells were then centrifuged at 700*g* for 3 minutes before removing the supernatant and resuspending the pellet in fresh full medium. Subsequently, about 25% of the cell suspension was seeded in a new culture flask.

### Cargos

In NS-EP, the cargo is transported into the cells by an interplay of diffusion and electrophoretic forces. To easily assess the extent of electrophoretic transport in an electroporation experiment, the diffusive transport during the experimental time frame must be minimized, which calls for using large cargo molecules. PI has a radius of approximately 5 Å (ref. 23) and therefore diffuses across the nanostraw substrate (25 μm, which is the initial thickness of the PC membrane) in ≈0.7 s (see ESI 2† for details). In contrast, the pMAX GFP plasmid (3.5 kpb) has been reported to have a radius larger than 50 nm ^24^ and should not massively diffuse to the cytosol without the help of electrophoresis (*t* = 71 s for a 50 nm radius object to diffuse 25 μm). Therefore, for electrophoresis assessment, we used pMAX plasmid as cargo (prepared by the Cell & Gene Therapy Core at Lund Stem Cell Center). The choice of a large molecule also eliminates the contribution from spontaneous transfection seen for small molecules.^16^

If not stated otherwise, 0.2 μg μl^−1^ of plasmid in 0.1× DPBS was used as the cargo solution. The plasmids were labeled with the intercalating dye YOYO-1 iodide (1 mM solution, Thermo Fisher), which fluoresces 1000-fold more when bound to DNA. YOYO-1 was diluted to 100 μM in MilliQ (MQ) water and mixed by vortexing or pipetting, then added to the plasmid solution to a final concentration of one YOYO-1 molecule per 250 base pairs. The suspension was kept at 50 °C for 2 hours to ensure uniform staining of the plasmid. The transfection efficiency was assessed using flow cytometry, detecting green fluorescence in cells immediately after transfection using YOYO-1-stained plasmids. Controls, *i.e.*, cells injected with only YOYO-1, showed no fluorescence.

### Device assembly

The nanostraw substrate was attached to a plastic cylinder (4 mm in diameter and 1 cm in height) that functions as a cell reservoir, using double-sided adhesive tape (3M 8153LE (300LSE) double-lined Adhesive Transfer Tape), with the nanostraws facing the cylinder. The tape was pre-cut to a circular ring matching the cylinder cross section using a laser cutter (Epilog Laser Fusion M2). The excess nanostraw substrate outside the cylinder was trimmed away, and the devices were then sterilized with UV ozone for 2 minutes.

### NS-EP for cell transfection

**Fig. 2:**
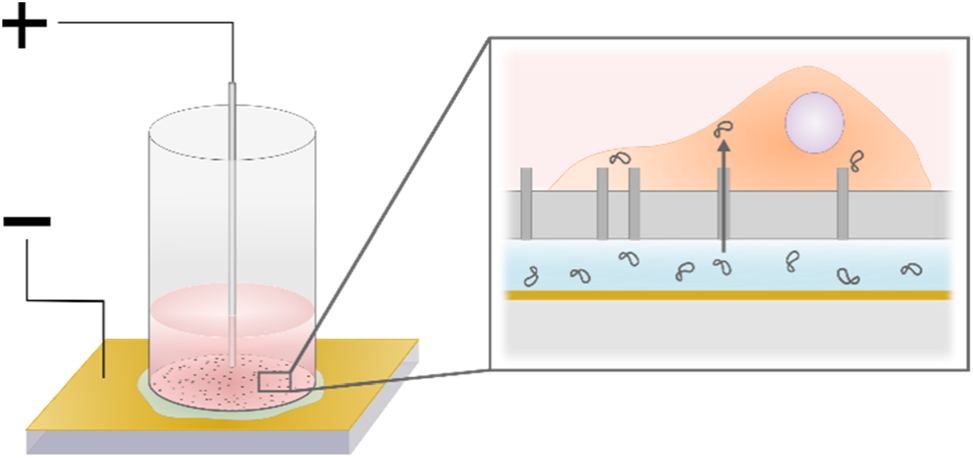
Schematics of the NS-EP method. Left: cell reservoir consisting of a cylinder with a nanostraw membrane at the bottom. Cells are spun down on the nanostraw membrane, which is placed on top of the cargo solution on a Au-electrode. A Pt-wire is inserted in the cell medium. Right: close-up showing cargo in solution underneath the nanostraw membrane and the direct cellular access obtained with NS-EP.

Cells were suspended in full medium according to the protocol described in Section 2.3, diluted ten times in DPBS, and then counted using flow cytometry (see gating in ESI 1†). The fraction of dead cells was measured using DAPI (Roche, Basel, Switzerland), a membrane-impermeable dye that binds to DNA. The nanostraw cylinders were placed in a 24-well plate with culture medium around them to keep the backside of the membrane wet. Each cylinder was filled with a specific number of cells (5000 for achieving a cell density of 385 cells per mm^2^, 20 000 cells for a cell density of 1540 cells per mm^2^, 35 000 cells for 2690 cells per mm^2^, 67 000 cells for 5150 cells per mm^2^, and 100 000 cells for 7690 cells per mm^2^) and topped up with medium to a total volume of 100 μl. The cylinders were then centrifuged at 200*g* for 1 min to spin down the cells on the nanostraws. Before electroporation, 70 μl of culture medium was removed from the cylinders to minimize the contact area between the cell medium and the top electrode. This step improved the control over the electrical pulses and the reproducibility of the results. The backside of the substrate was dried off, and the cylinder was placed on the bottom electrode, a gold-coated glass slide (100 nm Au thickness, Platypus Technologies), on top of 15 μl of the cargo solution. The top electrode was dipped into the cell medium, in the center of the cylinder at an inter-electrode distance of 0.5 mm (Fig. 2). A pulse generator (TGP110, Aim and Thurlby Thandar Instruments, Huntingdon, UK) and an amplifier (WMA-300, Falco Systems BV, Katwijk aan Zee, Netherlands) controlled the electrical pulses, while an oscilloscope was used for monitoring. The NS-EP was performed by applying two series of 40 s long pulse trains (frequency of 40 Hz, pulse width of 200 μs), lifting the cylinder in between. After electroporation, the nanostraw device was dried on the backside with a tissue and placed back in culture medium.

In the case where nanopores were used (Fig. 7), the same electroporation protocol was used as for nanostraws. For simplicity, we use the same term NS-EP for the electroporation process taking place using nanopores. Each experiment also contained a triplicate of control samples, where the same number of cells as for the NS-EP samples were analyzed but without being in contact with the nanostraw substrate.

### Flow cytometry

For flow cytometry measurements, a MACSQuant Analyzer 16 flow cytometer (Miltenity Biotech, Bergisch Gladbach, Germany) was used together with MACSQuant running buffer, storage solution, and washing solution. For assessing cell concentration and viability before seeding the cells, the cell suspension was diluted ten times in DPBS containing DAPI (1 : 100 in volume of 10 μg ml^−1^ DAPI stock solution in MQ water) and analyzed using flow cytometry.

To analyze the transfection efficiency immediately after NS-EP, cells were resuspended by pipetting up and down immediately after NS-EP. Cells in each cylinder were analyzed separately in the flow cytometer after staining with DAPI (1 : 100 in volume, as described above).

### Evaluation of GFP expression and cell count 48 h after NS-EP

After NS-EP, cells were detached from the substrate by pipetting them up and down. One-third of the total volume was measured using flow cytometry, from which the cell concentration and the total amount of detached cells was estimated. We have verified that the number of detached cells immediately after NS-EP corresponds to the number of seeded cells (data not shown). From the measured cell concentration in each sample, 10 000 cells were re-seeded into a 48-well plate and cultured for 48 hours. After 48 hours, the cells were detached from the substrate using trypsin (rinsing cells with DPBS and adding 50 μl trypsin for 3 min) and re-suspended in 300 μl cell medium. Subsequently, 150 μl of each sample was analyzed using flow cytometry, yielding cell count, GFP fluorescence, and cell death (using DAPI as described above). The cell count was multiplied by 2 to estimate the total amount of cells present after 48 hours. Control and mock experiments were performed. The control cells were seeded in 48-well plates with no contact with the nanostraw/nanopore substrate prior to flow cytometry analysis. The mock samples were subjected to NS-EP treatment using nanopores, without plasmids, that is, injection of MQ only.

### Simulations

The electrostatic simulation of the nanostraw membrane was done in COMSOL Multiphysics V6.0 using the electric current (EC) module. The simulation parameters and the model were adjusted to reflect the experimental conditions as much as possible. The cell membrane-nanostraw interaction is very complex and heterogeneous. To reduce complexity, we considered the cell as an oval cap sitting flat on top of 1 μm long nanostraws. The simulations were performed on a thin cross-sectional cell slice to lower the computational burden. The slice-thickness was taken as half the calculated nanostraw pitch, assuming a square array of nanostraws. As a nanostraw device is placed on the droplet of cargo solution, there will be some mixing between the cell medium, which is present on the top and in the straws, and the cargo solution. We assumed that the cargo solution was uniformly mixed with the cell medium in the nanostraws and approximated the conductivity of the mixture to the volume-weighted average of the two solutions' conductivity (see ESI 3†).

### Statistics

To investigate the effects of applied voltage, cell density, and nanostraw *vs.* nanopore, at least three independent experiments (*n* ≥ 3), each in triplicates, were performed for each sample condition, and the mean for each experiment was calculated. For the conductivity experiments, at least three independent experiments (*n* ≥ 3) were performed, however, not always in triplicates. In that case, the mean value of each single/duplicate/triplicate experiment was first calculated. For the bar plots, the mean values were averaged and the standard error was calculated. A one-way ANOVA with a Tukey Post Hoc test was performed on all data sets with *p < 0.05, **p < 0.01, and ***p < 0.001, except for transfection and intensity of the nanostraw/nanopore experiments where a *t*-test was performed instead.

## Results and discussion

### Effect of voltage on transfection efficiency

In the first set of experiments, we transfected cells with the pMAX GFP plasmid in 0.1× DPBS, stained with the YOYO-1 fluorescent intercalating dye (see methods for detailed protocol). The cell density for these experiments was 5150 cells per mm^2^. We applied two square electrical pulse trains of various amplitudes (from 14 V to 36 V) of pulse width 200 μs, applied at 40 Hz for 40 s across the nanostraw substrate. The cells were then pipetted off the substrate, labeled with the fluorescent dye DAPI (indicating dead cells), and analyzed using flow cytometry. Cells positive for DAPI and YOYO-1 fluorescence correspond to dead and transfected cells, respectively.

Cell transfection efficiency is reflected in the combined assessment of the percentage of cells transfected, the percentage of live cells, and the YOYO-1-fluorescence intensity of the transfected cells.

**Fig. 3:**
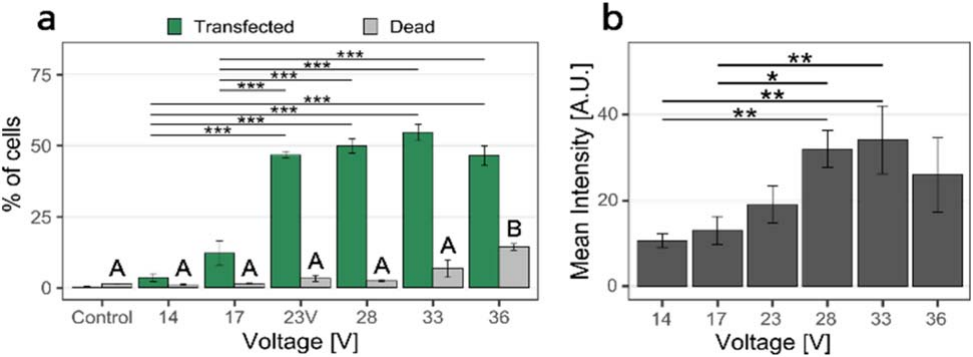
NS-EP transfection efficiency and cell viability as a function of applied voltage. (a) Proportion of transfected cells (*i.e.* cells positive for YOYO-1 fluorescence, mean percentage, and standard error, green) and proportion of dead cells (*i.e.* cells positive for DAPI fluorescence, % and standard error, gray). (b) Fluorescence intensity of transfected cells (mean value and standard error). The percentage of transfected cells was calculated relative to the population of live cells, see ESI 1† for gating strategy. The proportion of transfected cells and their fluorescence intensity increased with increasing the voltage, up to 28–33 V, and decreased for 36 V. The proportion of dead cells was low (<5%) for applied voltages between 14 and 28 V and increased slightly for an applied voltage of 33 V. At 36 V applied voltage, the number of dead cells increased to >14%. The cell density was 5150 cells per mm^2^. The difference between data labeled A and B is significant, with *p <* 0.05. (*n* = 3, the statistics were calculated with ANOVA and Tukey Post Hoc test: ***p < 0.001, **p < 0.01, *p < 0.05).

Increasing the voltage from 14 V to 28 V resulted in an increased proportion of transfected cells and higher fluorescence intensities (Fig. 3). However, increasing the voltage further, from 28 V to 33 V, resulted in higher cell death without any significant increase in the proportion of transfected cells nor in intensity. At 36 V, the transfection efficiency decreased while the proportion of dead cells increased further. Therefore, we chose 28 V as the electrical pulse amplitude in further experiments.

### Effect of cell density

**Fig. 4:**
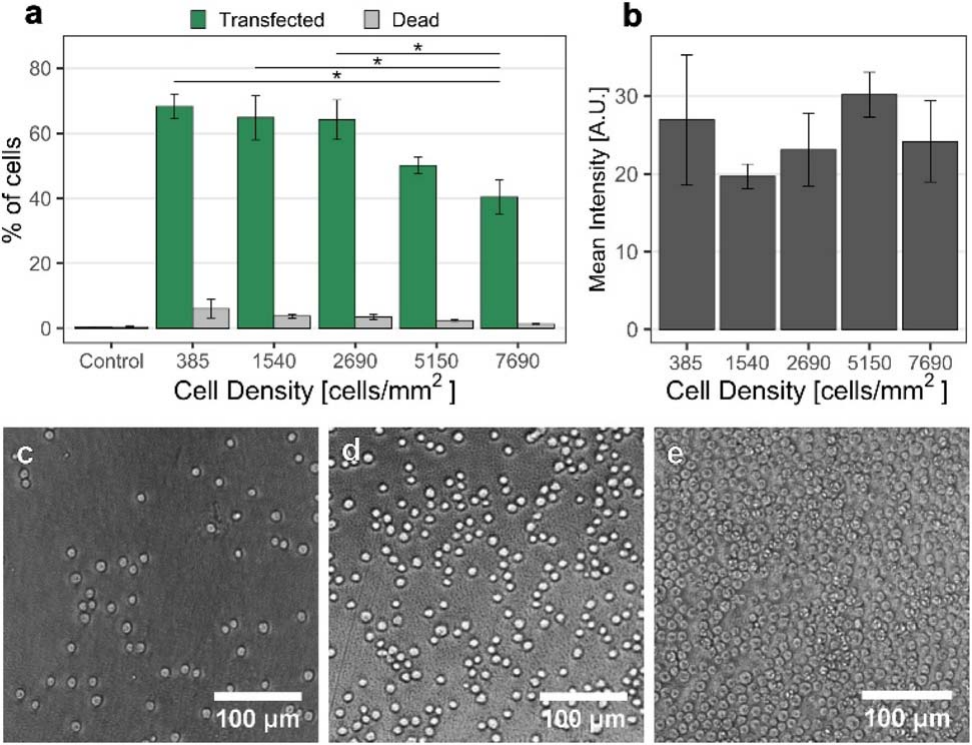
Effect of cell density on NS-EP transfection efficiency. (a) Proportion of transfected cells (mean % of cells and standard error). (b) Mean plasmid fluorescence intensity of transfected cells for the tested cell densities (mean value and standard error). The transfection efficiency increased with decreasing cell density and reached a plateau at 2690 cells per mm^2^. The fact that the percentage of transfected cells did not increase at higher cell densities was attributed to cell stacking, as can be seen in (e) (*n* = 3, ANOVA and Tukey Post Hoc test, *p < 0.05). No significant difference in intensity between the different densities could be identified. (*n* = 3, ANOVA and Tukey Post Hoc test). (c)–(e) Bright-field images of cells on nanostraw substrates, seeded at a cell density of 385, 2690, and 7690 cells per mm^2^, respectively. In (e), cells are stacked on top of each other in certain areas, while in (c) and (d), all cells are interfacing the nanostraws.

Another parameter possibly influencing the transfection efficiency is the cell density on the nanostraw substrate. Therefore, we have assessed pMAX transfection using a 0.5 mm inter-electrode distance and applying 28 V for cell densities varying from 385 cells per mm^2^ to 7690 cells per mm^2^ (Fig. 4).

The results show a lower proportion of transfected cells at high cell densities. One explanation could be the frequent occurrence of cell stacking at these densities, resulting in many cells not interfacing any nanostraws. This is supported by the microscopy images of the cells on the nanostraw substrate at high cell density, clearly showing stacked cells in multiple locations on the substrate (Fig. 4e). The fluorescence intensity of transfected cells did not vary significantly for different cell densities (Fig. 4b), which suggests that, on average, cells are transfected to the same extent, independently of the cell density, as long as they can interface the nanostraw substrate. This implies that the electrophoretic forces responsible for transporting the cargo are not affected by the cell density. To maximize the transfection efficiency and scalability of the method, we decided to work with a cell density of 2690 cells per mm^2^.

### Effect of the cargo solution conductivity

We used COMSOL simulation to theoretically evaluate the effect of cargo solution conductivity on the NS-EP performance. The simulation parameters and assumptions can be found in ESI 3.† The results are summarized in Table 1, presented as the voltage drop across the cell membrane positioned directly on the nanostraw before pore opening and the voltage drop across the nanostraw after NS-EP.

**Table 1:** COMSOL simulation results for the voltage drop across the cell membrane before forming pores in the cell membrane and the voltage drop across the nanostraw substrate after membrane pore formation for an applied voltage of 28 V (0.5 mm interelectrode distance) and different cargo solution conductivities (which were measured experimentally)

Cargo solution	Conductivity *σ* [mS cm^−1^]	Voltage drop across the cell membrane before pore formation	Voltage drop across nanostraw substrate after cell membrane pore formation
10× DPBS	78.000	23.46	1.48
DPBS	13.200	26.84	7.58
Cell medium	11.800	26.92	8.22
0.1× DPBS	1.690	27.32	20.48
0.01× DPBS	0.175	25.37	26.19
0.0025× DPBS	0.054	22.24	26.79
MQ water	0.001	14.49	27.06

For the transfection to be successful, one needs to open the cell membrane and drive the plasmid across the nanostraw substrate to the cytosol using electrophoresis. Membrane poration happens when a voltage drop on the order of 1 V occurs across the cell membrane.^25^ For an applied voltage of 28 V, the simulations show that this happens in all tested configurations (Table 1).

**Fig. 5:**
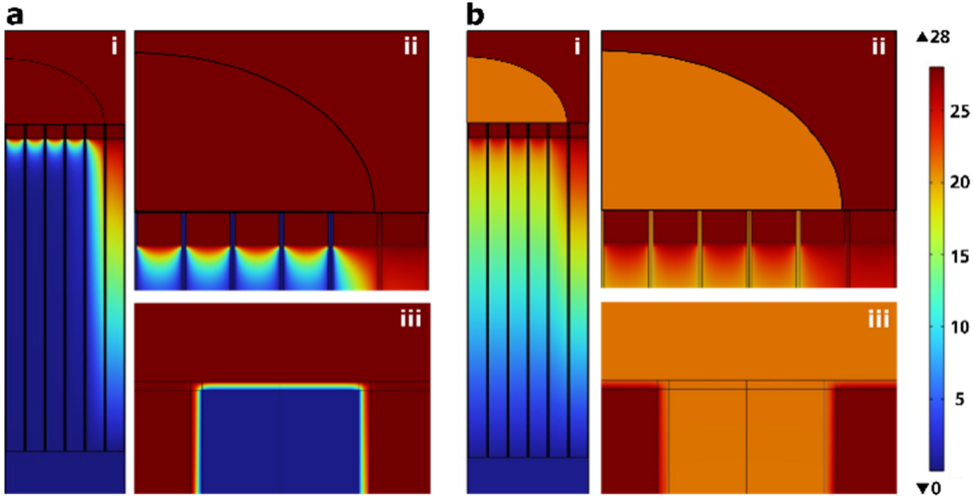
Voltage drop in the nanostraw device interfacing a cell (pictured as half an oval cap here) in the case where 0.025× DPBS is used as cargo solution, (a) before and (b) after pores have formed in the cell membrane. The cell sits flat on top of 1 μm high nanostraws and is shown at three different magnifications: (i) cell and nanostraw substrate, (ii) close-up view of the cell and nanostraws right under, (iii) close-up view of the nanostraw-cell membrane interface with only one nanostraw visible. The images show a side view of the 3D simulation of a thin slice.

Fig. 5 shows an example, for a cargo solution conductivity of 0.054 mS cm^−1^, where Fig. 5a and b depict the voltage drop across the device before and after pore formation in the cell membrane, respectively. In Fig. 5a, the voltage drop occurs mainly on the cell membrane (see Fig. 5a-iii), while after the pores have opened (Fig. 5b), the voltage drop occurs primarily across the nanostraw substrate (see Fig. 5b-i).

**Fig. 6:**
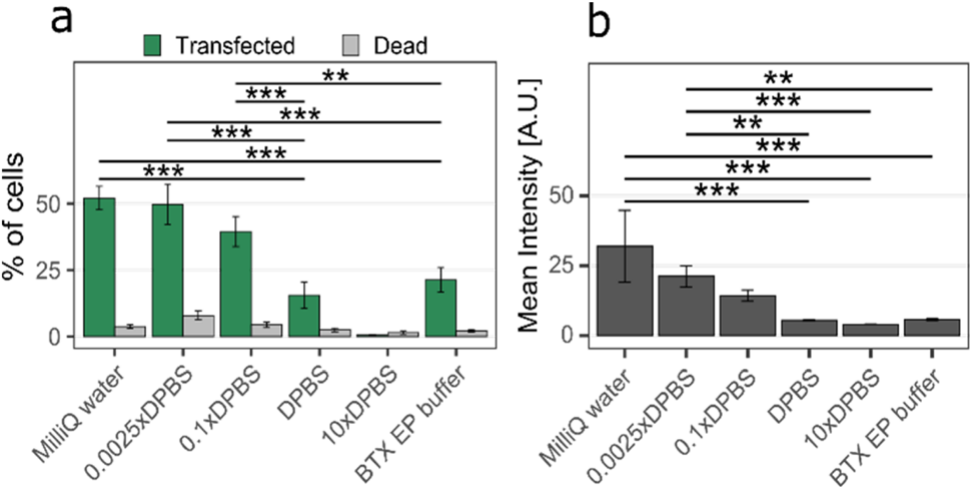
The effect of cargo solution conductivity on (a) the proportion of transfected cells and the proportion of dead cells (mean % and standard error) and (b) the intensity of transfected cells (mean value and standard error). The highest transfection efficiency was achieved for MQ water. In contrast, 10× DPBS yielded significantly worse transfection efficiency than all other buffers tested (*p* < 0.001). (*n* ≥ 3, ANOVA and Tukey Post Hoc test: ***p < 0.001, **p < 0.01, *p < 0.05).

Parameter optimization should aim to achieve efficient electrophoretic transport of cargo to the cytosol, therefore maximizing the voltage drop along the nanostraws after cell membrane pore formation, without detrimental effects on the cell viability. The simulations show that MQ water would result in the highest voltage drop across the nanostraws. We performed experiments to measure the electrophoretic transport of pMAX plasmids while simultaneously measuring the cell viability for various cargo solution conductivities. The experiments included buffer conductivities ranging from 0.001 mS cm^−1^, corresponding to MQ water, to 78 mS cm^−1^, equivalent to 10× concentrated DPBS. A commercial electroporation buffer (BTXPress, Fisher) was also included in the test. Other experimental parameters were chosen according to the optimum values presented above, *i.e.* optimum cell density (2690 cells per mm^2^) and applied voltage (28 V, except for 10× DPBS, where it was set to 20 V as the amplifier could not maintain 28 V due to the high conductivity). The maximum percentage of cells transfected occurred for MQ water, which also corresponds to the highest average YOYO-1 intensity in cells (Fig. 6), indicating a greater amount of cargo in the positive cells in comparison to other buffer conductivities. In contrast, using 10× DPBS resulted in the lowest proportion of transfected cells and lowest intensity, with close to 0% transfected cells. This can be explained by both the lower applied voltage (20 V instead of 28 V) and the lower voltage drop across the nanostraw substrate after cell membrane pore formation for these high conductivities (see Table 1). However, according to Fig. 3, at similar voltages, 0.1× DPBS cargo solution results in significant transfection efficiency (13% and 48% for 17 V and 23 V, respectively). Therefore, we can conclude that the main contribution to the low efficiency reported for 10× DPBS is the low voltage drop across the substrate, which hinders the electrophoretic transport of the plasmid. The cell viability remained high (>90%) for all cargo solution conductivities.

### Nanostraws compared to nanopores

Nanostraws were compared to nanopores with respect to transfection efficiency and cell viability. The nanopores were made by coating the PC membrane with the same thickness of alumina as the nanostraws using ALD and not processing them further. For these experiments, the transfection efficiency and cell death were first assessed immediately after NS-EP by measuring the YOYO-1 fluorescence and 48 h after NS-EP by evaluating the GFP fluorescence.

**Fig. 7:**
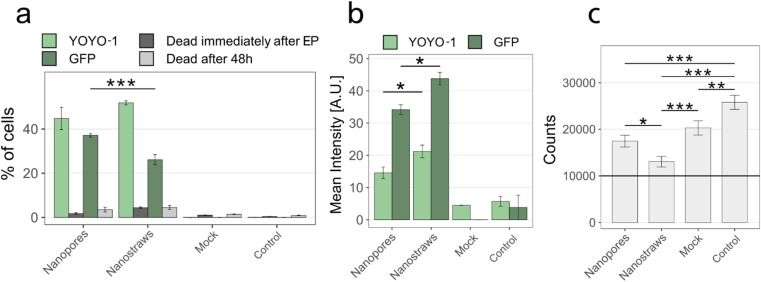
Effect of using nanostraws *versus* nanopores on transfection efficiency and viability, immediately- and 48 h after NS-EP. (a) Percentage of transfected cells immediately after EP (YOYO-1) and GFP expression after 48 hours (GFP), and cell viability at both time points. (b) Mean fluorescence intensity of the transfected cells shown in panel a. Note that the flow cytometry gains used for the mean intensity measurements are different for YOYO-1 and GFP since GFP has a much stronger fluorescence than YOYO-1. Therefore, the intensity values of YOYO-1 and GFP cannot be compared. The non-zero intensity in mock and control is due to auto-fluorescence and to a few cells appearing above the gating threshold (see ESI 1†). (c) Cell count for all conditions 48 hours after NS-EP. To assess the cell count after 48 h, 10 000 cells (black horizontal line) were seeded in a 48-well plate immediately after NS-EP, cultured for 48 h, and then counted. For all panels, controls denote cells cultured in a 48-well plate, without being in contact with nanostraws or nanopores. “Mock” are cells that are subjected to NS-EP but injected with only MQ water without cargo molecules. (*n* ≥ 3, MQ was used as buffer, error bars indicate standard error. (a) and (b) *t*-Test, (c) ANOVA and Tukey Post Hoc test: ***p < 0.001, **p < 0.01, *p < 0.05).

The results can be seen in Fig. 7. There is no significant difference in the percentage of transfected cells immediately after NS-EP between nanopores and nanostraws (Fig. 7a, YOYO-1). However, for the cells successfully transfected, the YOYO-1 fluorescence intensity was higher for nanostraws than nanopores. This suggests a higher amount of plasmids being transported to the cytosol using nanostraws. This is in discrepancy with the significantly lower percentage of GFP-expressing cells observed using nanostraws 48 h after NS-EP (Fig. 7b, GFP). There are two possible explanations for this. The first one is that cells transfected by nanostraws die to a larger extent. Indeed, even though there is no significant difference in the percentage of dead cells measured with flow cytometry between nanotraws and nanopores at the different time points (Fig. 7a), cells could detach from the substrate before the 48 h time point, which would make them invisible to flow cytometry dead cell count. The hypothetically larger amount of dead cells when using nanostraws could be due to the larger amount of plasmids transported to the cytosol. Cell death could be induced by the plasmids themselves as they have been shown to be cytotoxic,^20^ but also by the resulting higher number of GFP expressed in a cell as there are also indications that GFP is cytotoxic.^26^ The second possible explanation is that cells proliferate less after NS-EP using nanostraws compared to nanopores. Indeed, a recent study has shown that plasmids enter the nucleus mainly during the telophase.^27^ A lower rate of cell proliferation would, therefore, result in fewer plasmids entering the nucleus and lower GFP expression. Both possible explanations are supported by the significantly lower cell count obtained 48 h after NS-EP was performed using nanotraws compared to nanopores (Fig. 7c). However, with our experimental setup, it is not possible to assign the lower cell count to either proliferation issues or increased cell death. It is important to note that in comparison to the control, all NS-EP conditions (even the mock condition, where only buffer is transported to the cytosol) resulted in a lower cell count after 48 h. This effect is lower for nanopores than for nanostraws, which suggests that under the same electroporation conditions, nanopores are milder to cells than nanostraws.

Our and other's results suggest that using nanostraws and nanopores for cell transfection results in high efficiency and low toxicity. Whereas the cost of fabrication of nanostraws/pores can be significant, it can be minimized by using large PC membranes, thereby reducing the nanofabrication cost per area. Another advantage of using nanostraws/pores is the low amount of reagents necessary for transfection and the fact that the process does not require biosafety 2 laboratories, as opposed to when viral vectors are used. Therefore, depending on the application, it can be advantageous, not only from a scientific point of view but also for cost-effectiveness purposes, to use nanostraws and nanopores for cell transfection.

## Conclusions

In this work, we demonstrate the efficient transfection of clonal beta cells using NS-EP. We investigated the effects of EP voltage, cell density, cargo conductivity, and nanostraws *vs.* nanopores on the NS-EP transfection efficiency to further optimize the method. In terms of voltage effects, for a fixed inter-electrode distance, the transfection efficiency increased with voltage up to 28 V, while for higher voltages, the cell viability decreased. Regarding cell density, the transfection efficiency was constant at low cell densities, while for higher cell densities, it decreased with increasing density. This was attributed to cell stacking, which led to a subset of cells not interfacing with the nanostraws. Both simulations and experiments showed that MQ water is the best cargo solution for high viability and transfection efficiency. Simulations showed that, whereas all tested buffers yielded enough voltage drop over the cell membrane to open pores in the cell membrane at 28 V, MQ water led to the largest voltage drop across the nanostraw substrate after pore formation, thereby indicating a more effective electrophoretic transport through the nanostraws. Lastly, nanopores and nanostraws were compared, with cell viability and transfection efficiency evaluated both immediately and 48 hours after NS-EP. The results showed that although nanostraws provide a higher initial transfection efficiency, there were fewer cells and a lower percentage of GFP-expressing cells after 48 hours compared to when using nanopores. This could be attributed to either higher cell death, possibly caused by plasmid and/or GFP-toxicity, or a lower cell division rate after being interfaced with nanostraws, resulting in plasmids not entering the nucleus, preventing transcription. Since nanopores require less processing, it is also a more cost-efficient alternative to nanostraws. Together, these findings add to the understanding of NS-EP and contribute to increasing the usability of the method to beta cells, with a plethora of applications in diabetes research.

## Data availability

The data is available from the corresponding author upon request.

## Conflicts of interest

There are no conflicts to declare.

## Supplementary Material

RA-014-D4RA02791D-s001
