# Supplementary material for: Achieving efficient clonal beta cells transfection using nanostraw/nanopore-assisted electroporation
Source: RSC Adv. 2024 Jul 15;14(31):22244–52. doi: 10.1039/d4ra02791d (PMC11247384; doi:10.1039/d4ra02791d)
Supplement: RA-014-D4RA02791D-s001 [file RA-014-D4RA02791D-s001.pdf]

Electronic Supplementary Material (ESI) for RSC Advances.
This journal is © The Royal Society of Chemistry 2024

SUPPLEMENTARY INFORMATION

Achieving Efficient Clonal Beta Cells Transfection using Nanostraw/Nanopore-Assisted Electroporation.

Frida Ekstrand[1±], Mokhtar Mapar[1±], Sabrina Ruhrmann[2], Karl Bacos[2], Charlotte Ling[2], Christelle N. Prinz[1*]

[1]Division of Solid State Physics and NanoLund, Lund University, 221 00 Lund, Sweden

[2]Epigenetics and Diabetes Unit, Lund University Diabetes Centre, Department of Clinical Sciences, Scania University Hospital, 214 28, Malmö, Sweden

± These authors contributed equally to this work.

Correspondence to Christelle Prinz: christelle.prinz@ftf.lth.se

SI 1 Flow cytometry gating

Figure SI 1 show the gating strategy for non-transfected cells and (A) and (B), and for cells injected with stained plasmid in (C) and (D). Previously, the aggregates and debris have been filtered out of the data. (A) and (C) show the dead cells in a not-gate, so that only live cells are used for further analysis. Lastly, in (B) and (D), the live cells positive for YOYO-1 is displayed.

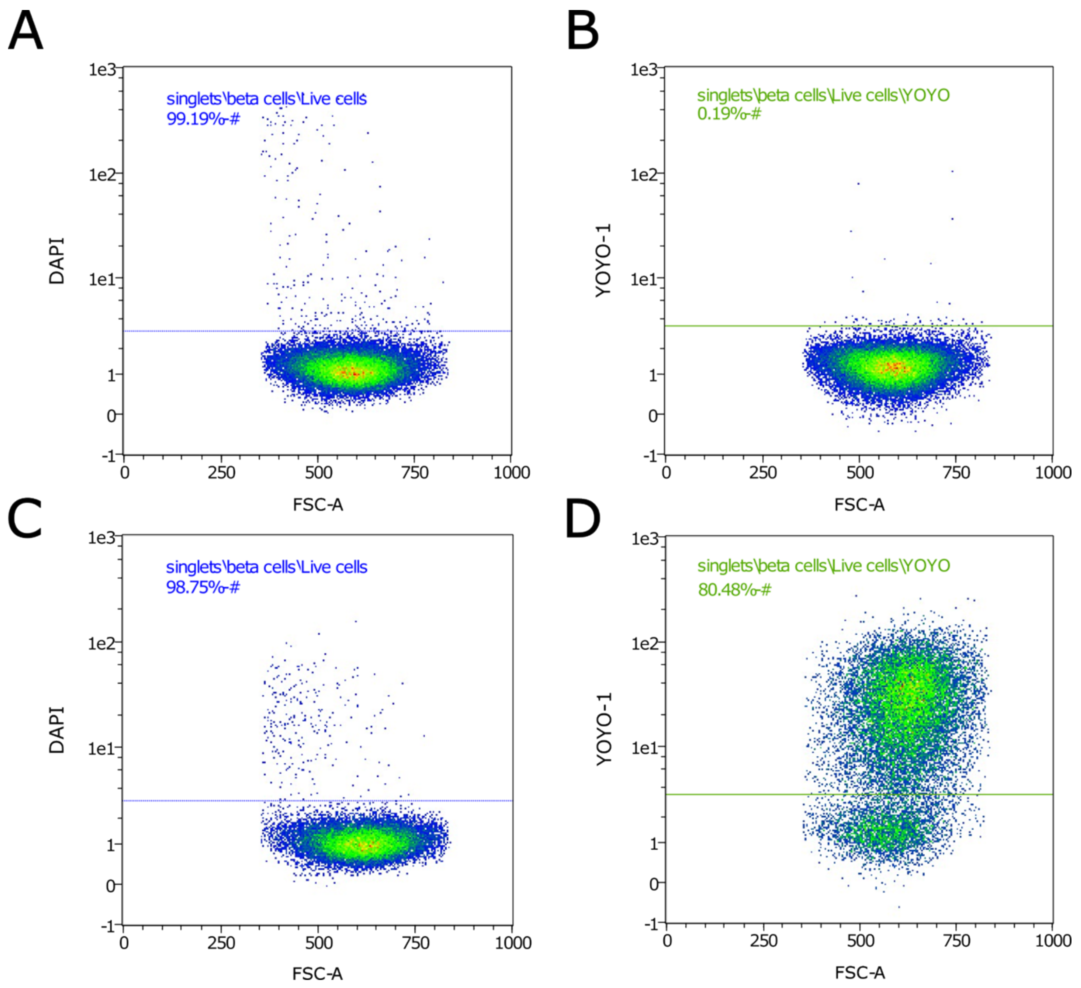

Figure SI 1: Flow cytometry gating is shown for non-transfected cells (A and B), and cells transfected with YOYO-1 stained plasmid (C and D). Aggregates and debris have been removed prior to these selections and dead cells are removed with a not-gate in (A) and (C). From the live cells the YOYO-1 positive cells are shown in (B) and (D).

SI 2 Evaluation of the diffusion times across the nanostraw substrate

The nanostraw membrane is 25 μm thick with the channels going through being 160 nm in diameter. One can therefore approximate the diffusion to be one dimensional along l = 25 μm long channels between the cargo reservoir and cytosol. The average squared distance over which small molecules like PI diffuses during a time t is given by

$$\langle l^2 \rangle = 2Dt, \tag{1}$$

where D is the diffusion coefficient. Approximating PI to small spheres, the Stokes-Einstein equation says that D can be expressed as

$$D = \frac{\mathrm{k}T}{6\pi\eta r} \tag{2}$$

where η is the viscosity of the cargo solution, r is the radius of the cargo, k the Boltzman constant and T the temperature. Combining equations 1 and 2 gives

$$l = \frac{\langle l^2 \rangle}{2D} = \frac{3\pi\eta r \langle l^2 \rangle}{kT}$$

which result in $t \approx 0.7$s when PI (r = 5 Å) diffuses in water at a temperature of 20 ° C.

SI 3 Simulation assumption and parameters

We made the assumption that the cell medium trapped in the pores was fully mixed with the analyte (cargo) solution underneath the nanostraw device and its conductivity was approximated by weighted averaging, i.e.

$$\sigma_w a = \frac{(V_p \sigma_m + V_a \sigma_a)}{V_p + V_a},$$

where V_p is the volume of the pores, σ_m the conductivity of the cell medium, V_a is the volume of the analyte underneath the nanostraws and σ_a is the conductivity of the analyte solution. The parameters in the simulation were the following:

Nanostraw pore inner diameter=136 nm

Pore density = $2 \cdot 10^7$

PC membrane thickness = 25 μm

Distance from the gold electrode to the nanostraw substrate=100 μm ("depth of the cargo solution), which was measured experimentally.

Cell membrane thickness: 8 nm.

σ_m=11.8 mS/cm

σ_c(10×DPBS)=78 mS/cm

σ_c(DPBS)=13.2 mS/cm

σ_c(0.1×DPBS)=1.69 mS/cm

σ_c(0.025 ×DPBS)=0.054 mS/cm

σ_c(Milli Q water)=0.001 mS/cm

σ(PC)=10^{-14} S/m

σ(alumina)=10^{-14} S/m

σ(cell medium)=11.8 mS/cm

σ(cell membrane)=5.10^{-7} S/m

ϵ_r (MilliQ water)=80

ϵ_r (PC)=2,9

ϵ_r(cell membrane)=8

ϵ_r (alumina)=9,8
